# The intersection of health inequalities and COVID‐19: Evidence from National Health Insurance Big Data in South Korea

**DOI:** 10.1002/ajcp.70044

**Published:** 2026-01-11

**Authors:** Jaehyun Nam, Sarah Jiyoon Kwon, Wonik Lee, Eunji Kim

**Affiliations:** ^1^ Department of Social Welfare Pusan National University Busan South Korea; ^2^ Department of Social Welfare University of Seoul Seoul South Korea

**Keywords:** COVID‐19, health inequalities, social determinants of health

## Abstract

Health inequalities persist along lines of income and wealth, shaped by unequal access to healthcare, differences in health behaviors, and pre‐existing chronic conditions. The COVID‐19 pandemic further put families in Korea under health strain and worsened their health outcomes. This study investigates how pre‐existing socioeconomic disparities intersect with health outcomes. Using the national administrative health big data provided by the Korean National Health Insurance Service, we employ logistic regression models for 10,459,043 randomly sampled cases from a total of more than 34 million registered positive cases in the country. We specifically examine whether and to what extent individuals from lower income backgrounds with positive cases are more or less likely to experience negative health outcomes. Our findings reveal that individuals from higher income backgrounds are less likely to experience negative health outcomes compared to the lowest income group, including hospitalization (odds ratio [OR] = 0.45, confidence interval [CI] = 0.44, 0.46), severe illness (OR = 0.70, CI = 0.61, 0.81), and fatalities (OR = 0.40, CI = 0.38, 0.41). Furthermore, our subsample analyses based on various demographic characteristics consistently showed that individuals from higher income backgrounds face a lower risk of adverse health outcomes. These results indicate that disadvantaged individuals are disproportionately affected by the health crisis, deepening health inequities. This paper emphasizes the need for targeted public policies.

## Highlights

- Our study's use of unique administrative health insurance data allows us to minimize the wellknown bias associated with self‐reported health data from surveys.
- Individuals from higher income backgrounds with positive cases are less likely to experience negative health outcomes, including hospitalization, severe illness, and fatalities, compared to those from the lowest income group.
- These results indicate that disadvantaged individuals are disproportionately affected by the health crisis, which may exacerbate health inequities, and emphasize the urgent need for targeted public policies.

## INTRODUCTION

COVID‐19 affected everyone, but its consequences were unequal, heavily influenced by socioeconomic status (Bambra et al., 2020; Geranios et al., 2022; Long et al., 2022; Nam & Lee, 2020; Organisation for Economic Co‐operation and Development, 2022). While the virus initially seemed to “not discriminate,” growing evidence now shows disparities in hospitalizations and mortality reflect existing inequalities in chronic diseases and social determinants of health (Bambra et al., 2020; Islam et al., 2021; McGowan & Bambra, 2022; Mendenhall et al., 2022). As of mid‐2023, more than 34.6 million confirmed cases and approximately 35,600 deaths had been reported in South Korea (hereafter, Korea), representing one of the highest cumulative infection rates among Organisation for Economic Co‐operation and Development (OECD) countries (Korea Disease Control and Prevention Agency [KDCA], n.d.; World Health Organization [WHO], n.d.). Although the government's early and coordinated public health response effectively mitigated the initial surge, substantial disparities in infection and fatality rates persisted across different socioeconomic groups (Jeon et al., 2023; Oh et al., 2021).

These unequal consequences reflect broader socioeconomic inequalities and illustrate what is often described as a *syndemic*, in which COVID‐19 interacts synergistically with pre‐existing structural and social vulnerabilities to amplify health disparities in disadvantaged communities. Originally developed to explain how HIV manifested among inner‐city residents of Hartford, Connecticut, in the United States (Mendenhall et al., 2022), the syndemic framework provides a valuable lens for understanding how chronic diseases and social inequalities interact to compound health risks during the pandemic.

To further explain why such inequalities persist and reproduce over time, it is also useful to draw on Link and Phelan's (1995) Fundamental Cause framework, which identifies social conditions as fundamental causes of disease. This concept helps explain how disparities in access to flexible resources—such as income, education, power, and social connections—continue to shape health outcomes even as new diseases emerge. In particular, socioeconomic status plays a critical role in shaping health outcomes through mechanisms such as disparities in access to healthcare and its subsequent influence on health behavior. Wealthier individuals generally have longer life expectancies, while those with lower status often face shorter ones, higher risks of chronic diseases, and poorer health overall. Greater income inequality can further reduce life expectancy for vulnerable groups, even when physical conditions are the same (Chetty et al., 2016; Pickett & Wilkinson, 2015; Wilkinson, 2020). Understanding these dynamics, particularly how existing inequalities and COVID‐19 may exacerbate one another, is essential for addressing health disparities and promoting health equity. This will ultimately foster social justice so that all individuals, regardless of their socioeconomic status, have the opportunity to lead healthier lives.

While these theoretical frameworks illuminate the structural mechanism of inequality, empirical evidence remains limited on how the health impacts of the COVID‐19 pandemic intersect with the pre‐existing disparities in financial resources over time since the onset of the pandemic (Bambra et al., 2020; Islam et al., 2021; McGowan & Bambra, 2022). In the Korean context, socioeconomic conditions are closely linked to occupational, education, health, and regional inequalities that shape vulnerability to the pandemic (Jeon et al., 2023; Oh et al., 2021). For example, individuals with lower socioeconomic status are disproportionately employed in occupations with greater exposure risk and limited job security. Moreover, low‐income groups tend to have poorer baseline health conditions and less access to outpatient services or supplementary private insurance coverage. Regional inequalities further compound these disparities, as residents in rural and coastal areas face barriers to tertiary hospitals and specialized medical care, which may delay diagnosis and treatment.

Building on these theoretical insights, this study addresses the lack of empirical evidence on whether adverse health outcomes are disproportionately concentrated among specific populations, particularly among low‐income groups, and identifies the health disparities resulting from the COVID‐19 pandemic.

One key contribution of this study is the use of unique nationwide administrative health big data developed by the Korean National Health Insurance Service (KNHIS), linked to COVID‐19 data from the Korea Disease Control and Prevention Agency (KDCA) and cause‐of‐death information from the Microdata Integrated Service of Statistics Korea (MDIS). A significant advantage of using this administrative dataset is its comprehensive coverage of approximately one‐third of all virus‐positive cases in Korea from 2020 to 2023, including over 10 million randomly sampled positive cases out of more than 34 million confirmed cases nationwide during this period. It provides highly accurate and robust information on health behaviors, health outcomes, various chronic diseases, prescription histories, as well as virus‐specific details such as confirmation dates and vaccination history. Our study's use of the national administrative health data allows us to avoid concerns on the reliability of survey‐based, self‐reported health data as well as systematic missingness in income and demographic characteristics, because these administrative records are linked to verified income and asset information.

Another key contribution is the study's wide time frame, spanning from October 2020 to June 2023, allowing us to capture both initial health shocks and long‐term pandemic impacts. This time frame also accounts for emerging virus variants and their varying effects. The substantial sample size, combined with comprehensive health data and a broad study period, enables us to more precisely assess the impact of COVID‐19 on health outcomes and its interactions with pre‐existing inequalities, which may have further intensified health disparities in Korea.

## METHODS

### Data

Data were drawn from the Korea Disease Control and Prevention Agency‐COVID‐19‐National Health Insurance Service cohort (K‐COV‐N cohort), which includes over 10 million confirmed cases in the country from October 2020 to June 2023. This unique administrative health insurance dataset allowed us to minimize the well‐recognized issue of bias in self‐reported health data from surveys (Miller et al., 2021).

### Measures

Our dependent variables consisted of three variables based on the severity of the COVID‐19 cases. First, we observed whether patients with the virus were hospitalized or not. Patients who visited the hospital or hospitalized due to the COVID‐19 virus were identified by diagnosis code (B34·2, B97·2, U07·1, U10·1, U10·9) from the 8th edition of the Korean Standard Classification of Diseases and Mortality, which is an adapted version of the 10th edition of the International Classification of Diseases. Second, we identified severe cases of the COVID‐19 virus if patients required the following treatments: heated humidified high‐flow nasal cannula, artificial ventilation, and extracorporeal membrane oxygenation. Lastly, we observed death cases from the virus, measuring case fatality.

To investigate our research question regarding social determinants of health during the pandemic, we included income as a six‐level categorical variable in the model, ranging from medical aid recipients to quintiles 1 through 5. Income levels were determined by the amount each individual pays for national health insurance, which was based on their income and wealth. Those at the bottom of the income distribution in the country who qualify for public medical assistance did not pay for health insurance. Thus, individuals in the Q1 were considered to have higher income levels than those receiving medical aid. The unit of analysis in this study was a case, where the same individual who tested positive for COVID‐19 multiple times may be included multiple times in the model. We reported clustered standard errors by individual.

To account for the emergence of virus variants and their potentially heterogeneous impacts (Fernandes et al., 2022), we divided the study period into different stages based on the dominant virus as follows: (1) Stage 1 from October 2020 to July 24, 2021, (2) Stage 2 from July 25, 2021 to January 15, 2022 (Delta), (3) Stage 3 from January 16, 2022 to September 3, 2022 (Omicron), and (4) Stage 4 from September 4, 2022 to 2023. We accounted for potential variations by COVID‐19 stage by adding stage fixed controls.

### Analytical strategy

We employed a logistic regression model to examine how socioeconomic characteristics are associated with the odds of hospitalization, severe cases, and death due to the COVID‐19 pandemic as such,

(1) $$\text{logit}\left({\hat{Y}}_{p}\right)=\alpha +\;\beta_{1}{\left(\mathrm{Income}\right)}_{p}+X_{p}\Phi +\gamma_{s}+e_{p},$$

where ${\hat{Y}}_{p}$ is the dependent variable for patient *p* tested positive for the COVID‐19 virus. $\beta_{1}$ is the coefficient of our interest in this study, which measures the association between income and our health outcomes. *X* denotes observable individual covariates that may be associated with both individuals' income resources and health outcomes, including sex, age, employment status, an indicator for residing in a metropolitan area, disability status, an indicator for vaccination as of the dates of confirmed positive cases, and the presence of any comorbidities. Importantly, the dataset did not contain information on employment type or job characteristics. As a proxy for employment type, we leveraged health insurance type in the dataset. The Korean national health insurance system broadly includes two types of insurance: employer‐based and community‐based. For individuals with employer‐based insurance, we differentiated between those who are actually employed and those who are not employed themselves but have family members who are. Previous research, such as Lee et al. (2024), using the same dataset, did not account for this distinction, instead employing a simple dichotomy between employer‐based and community‐based insurance. This approach may cause confusion by grouping non‐employed individuals whose family members are employed with those who are themselves employed, thus placing them all under the same employer‐based insurance category and potentially introducing confounding into the analysis. To address this limitation, we created a binary variable indicating employment status, coding only those who are actually employed as “employed” and all others as “not employed.” $\gamma_{s}$ represents stage controls based on the dominant virus variants.

## RESULTS

### Descriptive statistics

Table 1 displays the descriptive statistics for the full sample of over 10 million confirmed COVID‐19 cases in Korea, spanning from October 2020 to June 2023. Of the full sample, approximately 4% were hospitalized, 0.13% developed severe symptoms, and the fatality rate was 1.27%.

**Table 1:** Descriptive statistics for the full sample.

	Mean/%
Health outcomes	
COVID‐19 hospitalization	3.90
COVID‐19 severe cases	0.13
COVID‐19 case fatality	1.27
Demographic characteristics	
Female	53.92
Age	
<65	85.69
65–84	12.57
85≤	1.73
Income	
Q0 (medical aid recipients)	2.57
Q1	14.42
Q2	14.48
Q3	17.40
Q4	22.18
Q5	28.96
Employment status (employed = 1)	37.38
Metropolitan area (metropolitan = 1)	71.49
Disability	4.50
Vaccination (as of confirmed date)	79.67
Any comorbidities^a^	34.91
Pandemic stages	
Initial stage (October 2020–July 24, 2021)	0.53
Delta (July 25, 2021–January 15, 2022)	1.62
Omicron (January 16, 2022–September 3, 2022)	76.05
Last stage (September 4, 2022–June 2023)	21.81
*N*	10,459,043

^a^ *N* = 5,863,258.

Female cases accounted for slightly more than half (54%) of the total sample. The average age of confirmed cases in our sample was 40 years. In our model, we categorized age into three groups to reflect potential positive associations between age and the virus's fatality (Levin et al., 2020): individuals under 65 (86%), those aged 65–84 (13%), and those 85 or older (2%). Regarding income, the top income group (Q5) constituted the largest share, representing approximately 29%, followed by Q4 (22%) and Q3 (17.4%). Around 3% of the sample belonged to the lowest income group, which does not pay health insurance premiums. Approximately 37% of the sample were employed. Additionally, 35% of the sample was reported to have at least one comorbidity. Finally, 76% of confirmed cases occurred during the period when Omicron was the dominant variant.

## MAIN RESULTS

Table 2 shows results from the logistic regression models for the full sample. Model 1 is our preferred model with a full set of observable characteristics. Model 2 includes interaction terms between income and COVID‐19 stages, which capture whether and how the differences in health outcomes across income groups changed over time as the pandemic progressed. Compared to those at the bottom of the income distribution, individuals with higher incomes had lower odds of experiencing all three negative health outcomes from the virus, controlling for other observable characteristics and stage fixed effects. Specifically, for hospitalization due to COVID‐19 in Model 1 in Table 2, the odds ratios (ORs) for higher income groups from Q1 through Q5 ranged from 0.45 (*p* < .001) to 0.55 (*p* < .001), suggesting that individuals with higher incomes had about half the odds of hospitalization compared to medical aid recipients who are at the bottom of the income distribution (the reference group). Additionally, those who tested positive for the virus and belonged to higher incomes (Q4 and Q5) had approximately 30% lower odds of developing severe symptoms compared to those in the lowest income group. Compared to medical aid recipients, the odds of death from the virus were statistically significantly lower for individuals with higher incomes, with the OR decreasing from 0.64 (*p* < .001) for Q1 to 0.40 (*p* < .001) for Q5 as income rises, indicating that higher income is associated with a significant reduction in case fatality.

**Table 2:** Results from logistic regression for the full sample.

	Hospitalization		Severe cases		Fatalities	
	Model 1	Model 2	Model 1	Model 2	Model 1	Model 2
	OR (95% CI)	OR (95% CI)	OR (95% CI)	OR (95% CI)	OR (95% CI)	OR (95% CI)
Income (ref.: Medical aid recipients)						
Q1	0.549*** (0.533,0.566)	1.520* (1.049,2.203)	0.846* (0.730,0.981)	1.656* (1.024,2.678)	0.644*** (0.616,0.673)	1.083 (0.651,1.804)
Q2	0.515*** (0.499,0.531)	1.381 (0.956,1.996)	0.748*** (0.642,0.872)	1.196 (0.727,1.969)	0.514*** (0.490,0.538)	0.560* (0.318,0.986)
Q3	0.488*** (0.474,0.503)	1.236 (0.857,1.782)	0.665*** (0.572,0.772)	1.268 (0.777,2.069)	0.476*** (0.455,0.498)	0.585 (0.339,1.010)
Q4	0.471*** (0.457,0.485)	1.334 (0.925,1.923)	0.690*** (0.598,0.797)	1.253 (0.778,2.018)	0.412*** (0.395,0.431)	0.519* (0.307,0.880)
Q5	0.450*** (0.437,0.463)	1.489* (1.034,2.146)	0.701*** (0.611,0.805)	1.284 (0.807,2.043)	0.397*** (0.381,0.413)	0.466[Link], ** (0.281,0.774)
Age (ref.: 65>)						
65–84	2.112*** (2.080,2.144)	2.112*** (2.081,2.144)	5.507*** (5.055,5.998)	5.511*** (5.060,6.002)	5.833*** (5.659,6.012)	5.832*** (5.659,6.012)
85≤	6.511*** (6.320,6.709)	6.495*** (6.304,6691)	16.47*** (14.358,18.897)	16.398*** (14.296,18.808)	42.530*** (40.931,44.193)	42.530*** (40.926,44.187)
Female	0.927*** (0.916,0.938)	0.927*** (0.916,0.938)	0.442*** (0.415,0.471)	0.442*** (0.415,0.471)	0.436*** (0.427,0.445)	0.436*** (0.427,0.445)
Employed	0.659*** (0.650,0.668)	0.658*** (0.649,0666)	0.503*** (0.465,0.545)	0.502*** (0.464,0.544)	0.339*** (0.329,0.350)	0.339*** (0.329,0.350)
Metropolitan area	0.615*** (0.608,0.623)	0.616*** (0.608,0.623)	1.299*** (1.208,1.396)	1.300*** (1.210,1.397)	0.758*** (0.743,0.774)	0.758*** (0.743,0.774)
Disability	2.128*** (2.089,2.168)	2.119*** (2.080,2.159)	2.365*** (2.181,2.565)	2.365*** (2.181,2.565)	1.879*** (1.830,1.928)	1.879*** (1.831,1.928)
Vaccination	0.290*** (0.285,0.295)	0.291*** (0.286,0.296)	0.105*** (0.097,0.114)	0.105*** (0.097,0.114)	0.306*** (0.295,0.317)	0.306*** (0.294,0.317)
Comorbidity	1.488*** (1.468,1.507)	1.488*** (1.468,1.507)	2.406*** (2.211,2.618)	2.403*** (2.209,2.616)	1.875*** (1.823,1.929)	1.875*** (1.822,1.929)
COVID‐19 Stage (ref.: Initial stage)						
Delta	0.240*** (0.230,0.251)	0.278*** (0.191,0.403)	2.429*** (2.185,2.700)	3.777*** (2.274,6.273)	2.521*** (2.209,2.876)	3.237*** (1.955,5.358)
Omicron	0.003*** (0.003,0.003)	0.010*** (0.007,0.014)	0.139*** (0.124,0.156)	0.318*** (0.199,0.508)	1.737*** (1.539,1.961)	2.239*** (1.420,3.530)
Last stage	0.004*** (0.004,0.004)	0.001*** (0.007, 0.014)	0.149*** (0.131,0.169)	0.205*** (0.120,0.353)	1.860*** (1.646,2.101)	2.243*** (1.419,3.547)
Income × COVID‐19 stage						
Q1 × Delta		0.833 (0.565,1.228)		0.496* (0.283,0.870)		0.549* (0.309,0.974)
Q1 × Omicron		0.331*** (0.228,0.480)		0.402*** (0.240,0.673)		0.583* (0.350,0.974)
Q1 × Last stage		0.404*** (0.278,0.588)		0.774 (0.425,1.409)		0.618 (0.369,1.035)
Q2 × Delta		0.937 (0.637,1.377)		0.751 (0.422,1.336)		0.839 (0.447,1.576)
Q2 × Omicron		0.338*** (0.234,0.490)		0.490[Link], ** (0.286,0.838)		0.897 (0.508,1.584)
Q2 × Last stage		0.402*** (0.277,0.583)		0.764 (0.407,1.437)		0.979 (0.552,1.736)
Q3 × Delta		0.988 (0.673,1.448)		0.614 (0.348,1.083)		0.749 (0.407,1.377)
Q3 × Omicron		0.357*** (0.248,0.516)		0.390*** (0.230,0.662)		0.792 (0.458,1.372)
Q3 × Last stage		0.433*** (0.299,0.628)		0.662 (0.357,1.226)		0.874 (0.503,1.519)
Q4 × Delta		0.888 (0.606,1.302)		0.632 (0.363,1.099)		0.746 (0.413,1.345)
Q4 × Omicron		0.316*** (0.219,0.456)		0.418*** (0.251,0.698)		0.782 (0.460,1.328)
Q4 × Last stage		0.392*** (0.271,0.568)		0.724 (0.399,1.312)		0.825 (0.484,1.407)
Q5 × Delta		0.742 (0.507,1.088)		0.673 (0.392,1.154)		0.967 (0.550,1.701)
Q5 × Omicron		0·274*** (0.190,0.396)		0.403*** (0.245,0.662)		0.829 (0.499,1.379)
Q5 × Last stage		0.326*** (0.225,0.471)		0.688 (0.387,1.223)		0.894 (0.536,1.492)

*Note*: Each column presents the results from a multivariate logistic model. The reference category consists of medical aid recipients, representing the lowest income group. Q1 through Q5 correspond to the first through fifth income quintiles, respectively. Controls include demographics and medical histories, such as biological sex, age, employment status, urbanicity, disability, vaccination (as of confirmed date), any comorbidities (such as hypertension, diabetes, stroke, heart disease, hyperlipidemia, and cancer), pandemic stage dummies, and regional dummies (metropolitan area). The analyses include 5,863,258 cases.

Abbreviations: CI, confidence interval; OR, odds ratio.

* *p* < .05;

** *p* < .01;

*** *p* < .001.

*Source*: Authors' own analysis of data from the 2020–2023 Korean National Health Insurance Service (NHIS).

Notably, age was a critical factor in predicting the odds of experiencing negative health outcomes from the virus, with its ORs increasing from hospitalization to death. For example, COVID‐19 patients aged 85 and older had approximately 16 times the odds of developing severe conditions (OR = 16.47, *p* < .001) and 43 times the odds of fatality compared to those younger than 65 (OR = 42.53, *p* < .001). Male patients were, in general, more likely to experience severe health outcomes from the virus compared to their female counterparts. Vaccination was found to be effective in reducing the odds of developing these adverse health outcomes. Additionally, having at least one comorbidity increased the odds of experiencing negative health effects from the virus.

**Figure 1:**
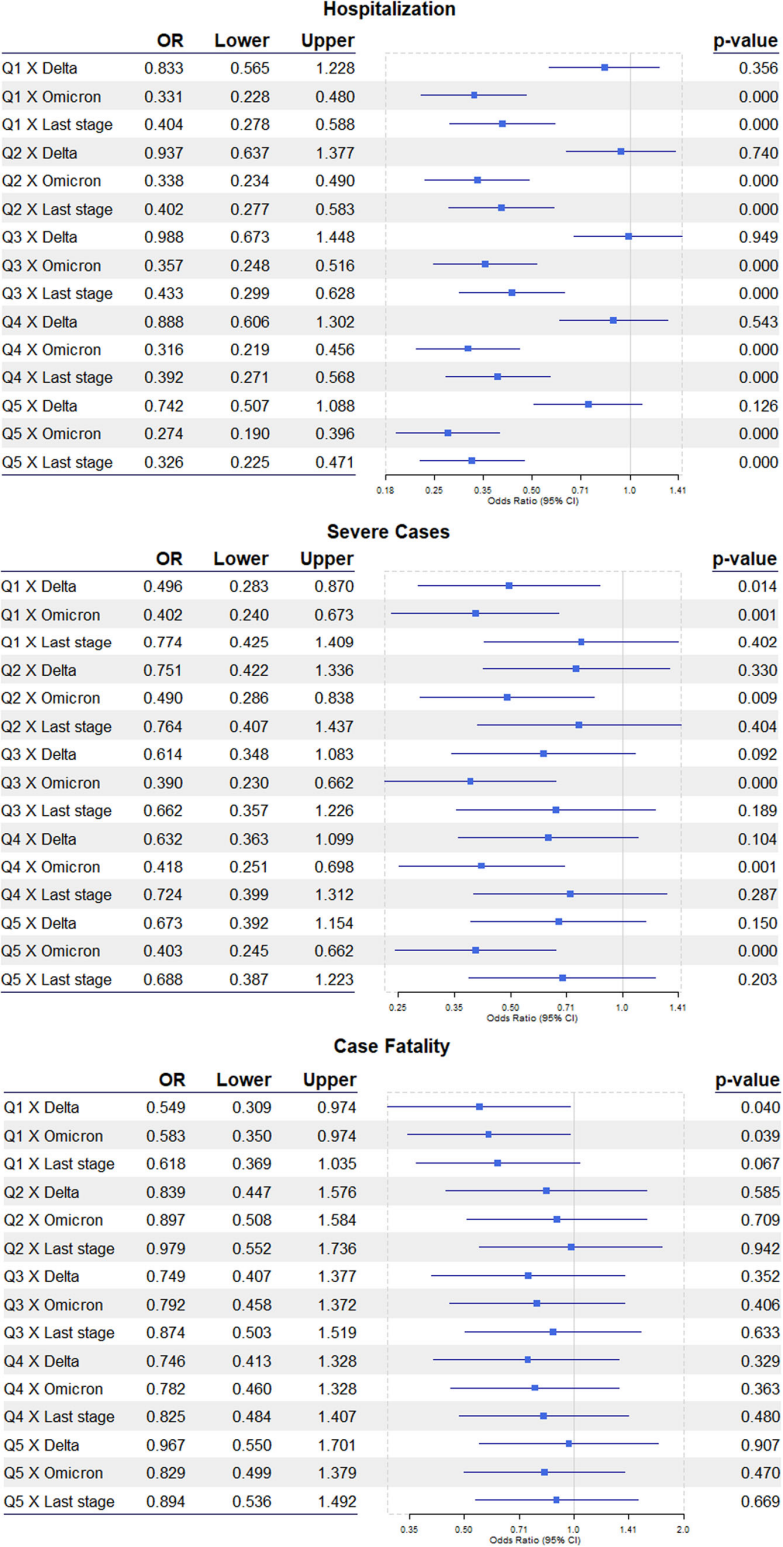
Interactions between income and pandemic stage on the odds of hospitalization rates, severe cases, and fatalities. *Note*: The reference category consists of medical aid recipients, representing the lowest income group. Q1 through Q5 correspond to the first through fifth income quintiles, respectively. Controls include demographics and medical histories, such as biological sex, age, employment status, urbanicity, disability, vaccination (as of confirmed date), any comorbidities (such as hypertension, diabetes, stroke, heart disease, hyperlipidemia, and cancer), pandemic stage dummies, and regional dummies (metropolitan area). The analyses include 5,863,258 cases. OR, odds ratio. *Source*: Authors' own analysis of data from the 2020–2023 Korean National Health Insurance Service (NHIS).

In Model 2 of Table 2, we included interaction terms between COVID‐19 stages and income groups to assess how, and to what extent, the associations between income and health outcomes changed as the pandemic progressed. Generally, the interaction terms for the later stages were statistically significant, in contrast to the early stages. For instance, as illustrated in the first panel of Figure 1, the ORs of 0.28 (*p* < .001) and 0.33 (*p* < .001) for the interaction between Q5 and the Omicron variant, as well as the final stage of the pandemic, respectively, for hospitalization, indicated that the negative associations between income and health outcomes became more pronounced in the later stages of the pandemic. The second and third panels present the interactions for severe cases and fatalities, respectively.

Next, we conducted subsample analyses by several demographic characteristics—sex, age, and employment status—to account for potential heterogeneity in the associations between income and health. First, Supporting Information S1: Tables S1 and S2 present the logistic regression results for female and male cases separately. Income was consistently negatively associated with all three adverse health outcomes from the virus for both female and male confirmed cases. Second, we examined whether the negative associations between income and health remained consistent when restricting the sample to individuals under 65 and those aged 65 and older, as shown in Supporting Information S1: Tables S3 and S4. Once again, income significantly predicted the likelihood of experiencing negative health outcomes for both groups. Finally, we disaggregated our sample by employment status in Supporting Information S1: Tables S5 and S6. Regardless of employment, individuals with lower incomes were more likely to experience severe illness from the virus.

## DISCUSSION

In this study, we examine the associations between socioeconomic disadvantages and health outcomes, and assess how pre‐existing inequalities interacted with the COVID‐19 pandemic from 2020 to 2023. Capitalizing on unique nationwide administrative health big data, we provide empirical evidence on the intersections of the pandemic and health outcomes. We build on the evidence of syndemic pandemics, where the synergistic interactions among the pandemic and pre‐existing inequality contribute to exacerbating health disparities. Our results from Korea offer strong evidence supporting the syndemic pandemics, demonstrating that the impact of COVID‐19 is intertwined with income inequalities, which have, in turn, widened health disparities in Korea.

The evidence from our results clearly shows that hospitalizations, severe cases, and fatality were predominantly observed among low‐income individuals. There is a growing body of empirical evidence demonstrating that morbidity and mortality vary across income levels, with income‐related health disparities increasing over time (Chetty et al., 2016; Khullar & Chokshi, 2018). There are several pathways through which health disparities by socioeconomic status have widened during the pandemic, explaining why the low‐income population was more vulnerable to the virus. This can be partly understood through the concept of syndemic pandemics. For instance, high‐skilled workers often have the option to choose their work environments, such as remote work, although some exceptions exist, such as in the case of healthcare workers. In contrast, low‐skilled workers generally lack this flexibility. Essential workers, especially hourly workers, including bus drivers, taxi drivers, cashiers, and delivery workers, had to continue working even during the peak of the COVID‐19 spread, which increased their risk of exposure to the virus.

Additionally, there is compelling evidence of a strong relationship between socioeconomic status and access to healthcare. During the pandemic, the number of patients receiving weekly treatment—measured against prior years—declined significantly, with this decrease being unevenly distributed and particularly pronounced among low‐income individuals (Frey et al., 2024). The pandemic has significantly impacted healthcare through various mechanisms, such as institutional practices that constrained healthcare supply, government interventions that transformed healthcare demand, and widespread behavioral changes in healthcare utilization, disproportionately affecting health outcomes across income classes.

In fact, Korea introduced temporary universal health coverage (UHC) for COVID‐19 at the beginning of the pandemic in 2020 and maintained it during the early stages (Lee et al., 2024). This initiative strengthened access to healthcare by eliminating out‐of‐pocket expenses for COVID‐19 diagnosis and treatment nationwide. However, as the pandemic prolonged and new variants of the virus, such as Omicron, emerged, patient numbers surged, leading to increasing healthcare costs that prompted the expiration of UHC. This, in turn, resulted in higher out‐of‐pocket expenses for testing, treatment, and medication, potentially limiting vulnerable individuals' access to healthcare and consequently, widening the health gap (El‐Khatib et al., 2020; Lee et al., 2024). During the early stages of the pandemic, the temporary UHC reduced financial barriers to care; however, as the pandemic progressed and the UHC expired, disparities in non‐fatal outcomes such as hospitalization and severe cases between income groups became more pronounced, suggesting that the increasing financial burden may have disproportionately affected low‐income individuals' access to timely and adequate care (Lee et al., 2021, 2024). In contrast, the association between income and COVID‐19‐related fatalities was significant during the initial stage but diminished over time, showing no consistent income‐related pattern in later stages. This may reflect improvements in vaccination coverage, which was implemented universally regardless of income level.

Meanwhile, age was found to be the strongest factor contributing to the health risk from the virus. While prior studies have indicated that sex, types of insurance, residential areas, presence of comorbidities, and disabilities are associated with a higher risk of hospitalization and death (Auger et al., 2023; Jeon et al., 2023; Lee et al., 2024; Theodore et al., 2023), our findings indicate that age was the most significant risk factor, followed by comorbidities such as hypertension, diabetes, stroke, heart disease, hyperlipidemia, and cancer. This aligns with results from the United States, from the current Centers for Disease Control and Prevention (CDC) reported that over 81% of COVID‐19 deaths occurred in individuals aged 65 and older, representing a risk 97 times higher than that of young adults aged 18–29 (Centers for Disease Control and Prevention, 2024). Future research should further examine the intersection between age, income, and health outcomes to better understand how multiple layers of social disadvantage may compound health risks during a pandemic. Additionally, vaccinations have played an important role in reducing the risk, as the COVID‐19 vaccine has proven to be highly effective at preventing serious illness, hospitalization, and death (Nordstrom et al., 2022; Richard et al., 2023). However, it should be noted that some evidence shows severe side effects associated with the vaccine, such as death, myocarditis, pericarditis, and Guillain–Barré Syndrome (Jeong et al., 2024; Laurini et al., 2023).

We acknowledge that our study includes several limitations. First, COVID‐19 cases may have been underreported since the data was based on insurance claims. While Korea conducted widespread screening during the early stages of the pandemic, testing became voluntary in the later stages, making it challenging to identify individuals who did not report their cases or seek medical care (Kang et al., 2020; Lee et al., 2024). Additionally, there was a social tendency to avoid acknowledging COVID‐19 infections, resulting in potential “shy” COVID‐19 positive individuals who did not report their cases. (Bhanot et al., 2020) Second, we ranked individuals' income levels based on their insurance premiums. This method is accurate for those with employer‐based health insurance, but not for those with community‐based health insurance, as the income information for local subscribers is often unreliable (Moon, 2021). Third, some cases in the KNHIS data are missing health history and comorbidity information. As a result, we excluded these cases, which account for 44% of the full sample. As a robustness check, we also tested the full sample without controlling for comorbidities, and the results remained qualitatively unchanged (results available upon request). Lastly, our employment status variable has limitations, primarily due to the gray area within the current national health insurance system (Lee et al., 2019; Park, 2023). Historically, employer‐based insurance was designed for individuals working in traditional, standard workplaces. However, with the rapid growth of nontraditional, nonstandard employment, some workers are not eligible for employer‐based insurance. For instance, freelancers are covered under community‐based insurance, which could lead to potential misclassification of workers in our coding scheme. Since our data does not provide detailed information on individuals' jobs and occupational characteristics, future research should explore how these nonstandard workers were impacted during the pandemic.

## CONCLUSION

Overall, our findings from the national administrative health big data confirm the intersections of income and health during the COVID‐19 pandemic for the full sample and across demographic groups, underscoring widening health disparities during the health crisis. This paper highlights the need for a comprehensive, multifaceted strategy to mitigate health inequalities by crafting policies that take into account individual health conditions and available resources. In particular, the findings can inform future public health and pandemic preparedness efforts by emphasizing equitable access to healthcare, financial protection for low‐income groups, occupational health for higher risk workers, and evidence‐based policy development to effectively reduce emerging disparities.

## CONFLICT OF INTEREST STATEMENT

The authors declare no conflicts of interest.

## ETHICS STATEMENT

This study was approved by the Institutional Review Board of the affiliated institution (IRB No. 2023_67_HR).

## Supporting information

Supporting information.

## Data Availability

This study uses restricted‐use data from the Korean National Health Insurance Service and therefore cannot provide the data publicly.
