# Supplementary material for: The intersection of health inequalities and COVID‐19: Evidence from National Health Insurance Big Data in South Korea
Source: Am J Community Psychol. 2026 Jan 11;77(3-4):485–95. doi: 10.1002/ajcp.70044 (PMC13289520; doi:10.1002/ajcp.70044)
Supplement: Supplementary file 1 — Supporting information. [file AJCP-77-485-s001.docx]

**ONLINE SUPPLEMENT**

**to article in**

*American Journal of Community Psychology*

**The Intersection of Health Inequalities and COVID-19: Evidence from National Health Insurance Big Data in South Korea**

**CONTENTS:**

**Appendix A: Results from logistic regression for the subgroups**

**Tables 1-6**

August 2025

Appendix Table 1. Results from logistic regression for female cases

|  | Hospitalization | | Severe cases | | Fatalities | |
| --- | --- | --- | --- | --- | --- | --- |
|  | Model 1 | Model 2 | Model 1 | Model 2 | Model 1 | Model 2 |
|  | OR  (95% CI) | OR  (95% CI) | OR  (95% CI) | OR  (95% CI) | OR  (95% CI) | OR  (95% CI) |
| Income (ref.: Medical aid recipients) |  |  |  |  |  |  |
| Q1 | 0.573***  (0.550,0.596) | 1.323  (0.796,2.196) | 0.939  (0.753,1.171) | 1.872  (0.880,3.984) | 0.732***  (0.687,0.781) | 1.617  (0.664,3.938) |
| Q2 | 0.539***  (0.518,0.562) | 1.238  (0.747,2.051) | 0.800  (0.633,1.011) | 1.745  (0.806,3.777) | 0.565***  (0.526,0.606) | 0.827  (0.303,2.258) |
| Q3 | 0.520***  (0.500,0.542) | 1.037  (0.626,1.715) | 0.685**  (0.544,0.863) | 1.348  (0.620,2.933) | 0.526***  (0.491,0.563) | 0.616  (0.225,1.688) |
| Q4 | 0.511***  (0.491,0.532) | 1.022  (0.617,1.693) | 0.696**  (0.558,0.868) | 1.222  (0.570,2.617) | 0.447***  (0.418,0.477) | 0.655  (0.253,1.694) |
| Q5 | 0.489***  (0.470,0.508) | 1.119  (0.676,1.854) | 0.683***  (0.554,0.843) | 1.122  (0.533,2.363) | 0.435***  (0.409,0.462) | 0.506  (0.200,1.282) |
| Age (ref.: 65>) |  |  |  |  |  |  |
| 65-84 | 2.045***  (2.004,2.087) | 2.041***  (2.001,2.083) | 4.572***  (3.981,5.250) | 4.586***  (3.993,5.268) | 5.849***  (5.569,6.144) | 5.848***  (5.568,6.143) |
| 85<= | 6.638***  (6.390,6.897) | 6.614***  (6.366,6.871) | 17.58***  (3.981,5.250) | 17.49***  (14.288,21.410) | 57.71***  (54.483,61.136) | 57.73***  (54.495,61.149) |
| Employed | 0.712***  (0.698,0.725) | 0.710***  (0.696,0.723) | 0.440***  (0.376,0.514) | 0.439***  (0.376,0.513) | 0.296***  (0.279,0.315) | 0.296***  (0.279,0.315) |
| Metropolitan area | 0.591***  (0.581,0.600) | 0.591***  (0.582,0.601) | 1.407***  (1.249,1.586) | 1.410***  (1.251,1.589) | 0.742***  (1.251,1.589) | 0.742***  (0.279,0.315) |
| Disability | 2.058***  (2.003,2.114) | 2.049***  (1.995,2.106) | 2.576***  (2.243,2.957) | 2.588***  (2.254,2.970) | 1.819***  (1.744,1.898) | 1.820***  (1.745,1.899) |
| Vaccination | 0.293***  (0.286,0.299) | 0.293***  (0.287,0.300) | 0.0964***  (0.086,0.109) | 0.0967***  (0.086,0.109) | 0.304***  (0.288,0.321) | 0.304***  (0.288,0.321) |
| Comorbidity | 1.501***  (1.474,1.528) | 1.500***  (1.474,1.527) | 2.638***  (2.290,3.039) | 2.633***  (2.285,3.033) | 1.889***  (1.804,1.978) | 1.888***  (1.803,1.978) |
| COVID-19 Stage (ref.: Initial stage) |  |  |  |  |  |  |
| Delta | 0.227***  (0.214,0.242) | 0.240***  (0.144,0.399) | 2.690***  (2.265,3.195) | 4.898***  (2.254,10.646) | 2.876***  (2.294,3.607) | 5.507***  (2.277,13.322) |
| Omicron | 0.00308***  (0.003,0.003) | 0.00710***  (0.004,0.012) | 0.144***  (0.120,0.173) | 0.311**  (0.149,0.649) | 1.952***  (1.587,2.402) | 2.794*  (1.227,6.362) |
| Last stage | 0.00369***  (0.003,0.004) | 0.00761***  (0.005,0.012) | 0.151***  (0.124,0.185) | 0.220***  (0.095,0.508) | 1.984***  (1.611,2.444) | 2.909*  (1.273,6.646) |
| Income X COVID-19 stage |  |  |  |  |  |  |
| Q1 X Delta |  | 0.920  (0.542,1.561) |  | 0.437  (0.185,1.033) |  | 0.304*  (0.115,0.807) |
| Q1 X Omicron |  | 0.399***  (0.240,0.664) |  | 0.416*  (0.185,0.934) |  | 0.462  (0.189,1.129) |
| Q1 X Last stage |  | 0.453**  (0.240,0.664) |  | 0.722  (0.185,0.934) |  | 0.439  (0.179,1.078) |
| Q2 X Delta |  | 0.986  (0.582,1.669) |  | 0.519  (0.216,1.245) |  | 0.431  (0.144,1.290) |
| Q2 X Omicron |  | 0.395***  (0.238,0.656) |  | 0.336*  (0.145,0.779) |  | 0.707  (0.258,1.937) |
| Q2 X Last stage |  | 0.449**  (0.269,0.747) |  | 0.461  (0.173,1.226) |  | 0.648  (0.235,1.784) |
| Q3 X Delta |  | 1.049  (0.620,1.775) |  | 0.478  (0.197,1.156) |  | 0.652  (0.219,1.942) |
| Q3 X Omicron |  | 0.466**  (0.281,0.772) |  | 0.407*  (0.176,0.944) |  | 0.859  (0.312,2.362) |
| Q3 X Last stage |  | 0.525*  (0.315,0.872) |  | 0.716  (0.275,1.864) |  | 0.865  (0.313,2.391) |
| Q4 X Delta |  | 0.990  (0.585,1.677) |  | 0.660  (0.279,1.561) |  | 0.457  (0.162,1.289) |
| Q4 X Omicron |  | 0.466**  (0.281,0.774) |  | 0.444  (0.195,1.007) |  | 0.695  (0.268,1.803) |
| Q4 X Last stage |  | 0.533*  (0.320,0.888) |  | 0.608  (0.237,1.561) |  | 0.669  (0.257,1.744) |
| Q5 X Delta |  | 0.831  (0.491,1.406) |  | 0.546  (0.234,1.273) |  | 0.750  (0.275,2.048) |
| Q5 X Omicron |  | 0.412***  (0.248,0.684) |  | 0.543  (0.245,1.203) |  | 0.861  (0.339,2.189) |
| Q5 X Last stage |  | 0.456**  (0.274,0.759) |  | 0.783  (0.317,1.935) |  | 0.866  (0.339,2.209) |

Source: Authors’ own analysis of data from the 2020-23 Korean National Health Insurance Service (NHIS). Notes: Each column presents the results from a multivariate logistic model. The reference category consists of medical aid recipients, representing the lowest income group. Q1 through Q5 correspond to the first through fifth income quintiles, respectively. The analyses include 3,263,866 cases. * p<0.05, ** p<0.01, *** p<0.001.

Appendix Table 2. Results from logistic regression for male cases

|  | Hospitalization | | Severe cases | | Fatalities | |
| --- | --- | --- | --- | --- | --- | --- |
|  | Model 1 | Model 2 | Model 1 | Model 2 | Model 1 | Model 2 |
|  | OR  (95% CI) | OR  (95% CI) | OR  (95% CI) | OR  (95% CI) | OR  (95% CI) | OR  (95% CI) |
| Income (ref.: Medical aid recipients) |  |  |  |  |  |  |
| Q1 | 0.504***  (0.481,0.529) | 1.725*  (1.004,2.964) | 0.792*  (0.649,0.967) | 1.572  (0.838,2.949) | 0.585***  (0.550,0.621) | 0.869  (0.462,1.636) |
| Q2 | 0.470***  (0.448,0.494) | 1.536  (0.900,2.622) | 0.727**  (0.593,0.891) | 0.960  (0.498,1.851) | 0.495***  (0.465,0.527) | 0.463*  (0.233,0.922) |
| Q3 | 0.446***  (0.426,0.468) | 1.597  (0.941,2.712) | 0.657***  (0.539,0.801) | 1.247  (0.660,2.356) | 0.455***  (0.429,0.484) | 0.561  (0.292,1.079) |
| Q4 | 0.428***  (0.409,0.448) | 1.911*  (1.127,3.240) | 0.690***  (0.570,0.836) | 1.287  (0.694,2.388) | 0.399***  (0.376,0.423) | 0.460*  (0.243,0.870) |
| Q5 | 0.411***  (0.393,0.429) | 2.169**  (1.281,3.673) | 0.719***  (0.599,0.864) | 1.390  (0.761,2.539) | 0.388***  (0.367,0.410) | 0.444**  (0.242,0.814) |
| Age (ref.: 65>) |  |  |  |  |  |  |
| 65-84 | 2.193***  (2.144,2.243) | 2.194***  (2.146,2.244) | 6.087***  (5.462,6.785) | 6.076***  (5.452,6.770) | 5.814***  (5.595,6.042) | 5.814***  (5.594,6.041) |
| 85<= | 6.364***  (6.063,6.680) | 6.372***  (6.070,6.688) | 14.18***  (11.680,17.208) | 14.18***  (11.683,17.205) | 29.17***  (27.637,30.788) | 29.17***  (27.635,30.786) |
| Employed | 0.616***  (0.604,0.628) | 0.613***  (0.601,0.626) | 0.530***  (0.482,0.582) | 0.527***  (0.480,0.579) | 0.348***  (0.335,0.361) | 0.348***  (0.335,0.361) |
| Metropolitan area | 0.647***  (0.636,0.659) | 0.648***  (0.636,0.660) | 1.243***  (1.135,1.361) | 1.245***  (1.137,1.363) | 0.785***  (0.764,0.806) | 0.785***  (0.764,0.806) |
| Disability | 2.167***  (2.111,2.225) | 2.153***  (2.098,2.210) | 2.261***  (2.046,2.498) | 2.258***  (2.043,2.495) | 1.906***  (1.844,1.969) | 1.906***  (1.844,1.969) |
| Vaccination | 0.285***  (0.278,0.293) | 0.287***  (0.279,0.294) | 0.112***  (0.100,0.124) | 0.112***  (0.101,0.125) | 0.311***  (0.296,0.327) | 0.311***  (0.296,0.327) |
| Comorbidity | 1.485***  (1.456,1.513) | 1.484***  (1.456,1.513) | 2.284***  (2.055,2.538) | 2.282***  (2.054,2.536) | 1.837***  (1.772,1.903) | 1.836***  (1.772,1.903) |
| COVID-19 Stage (ref.: Initial stage) |  |  |  |  |  |  |
| Delta | 0.255***  (0.239,0.272) | 0.343***  (0.200,0.588) | 2.272***  (1.987,2.599) | 3.006**  (1.519,5.949) | 2.325***  (1.977,2.734) | 2.249*  (1.557,2.103) |
| Omicron | 0.00325***  (0.003,0.003) | 0.0150***  (0.009,0.025) | 0.135***  (0.116,0.157) | 0.329***  (0.178,0.606) | 1.625***  (1.399,1.886) | 1.993*  (1.148,3.459) |
| Last stage | 0.00376***  (0.004,0.004) | 0.0135***  (0.008,0.023) | 0.147***  (0.125,0.173) | 0.199***  (0.097,0.407) | 1.809***  (1.557,2.103) | 1.933*  (1.109,3.371) |
| Income X COVID-19 stage |  |  |  |  |  |  |
| Q1 X Delta | 0.739  (0.419,1.305) |  | 0.560  (0.264,1.191) |  | 0.814  (0.393,1.682) |  |
| Q1 X Omicron | 0.268***  (0.156,0.462) |  | 0.379**  (0.193,0.747) |  | 0.639  (0.338,1.207) |  |
| Q1 X Last stage | 0.360***  (0.208,0.622) |  | 0.785  (0.354,1.739) |  | 0.744  (0.392,1.414) |  |
| Q2 X Delta | 0.885  (0.505,1.553) |  | 1.017  (0.469,2.207) |  | 1.261  (0.578,2.749) |  |
| Q2 X Omicron | 0.281***  (0.164,0.480) |  | 0.604  (0.299,1.221) |  | 1.004  (0.578,2.749) |  |
| Q2 X Last stage | 0.348***  (0.202,0.599) |  | 1.039  (0.453,2.384) |  | 1.248  (0.621,2.508) |  |
| Q3 X Delta | 0.878  (0.504,1.532) |  | 0.759  (0.358,1.613) |  | 0.876  (0.415,1.851) |  |
| Q3 X Omicron | 0.246***  (0.145,0.419) |  | 0.369**  (0.186,0.732) |  | 0.772  (0.400,1.490) |  |
| Q3 X Last stage | 0.326***  (0.190,0.556) |  | 0.609  (0.270,1.374) |  | 0.914  (0.400,1.490) |  |
| Q4 X Delta | 0.744  (0.427,1.295) |  | 0.665  (0.319,1.389) |  | 1.040  (0.503,2.153) |  |
| Q4 X Omicron | 0.192***  (0.113,0.326) |  | 0.392**  (0.203,0.760) |  | 0.833  (0.503,2.153) |  |
| Q4 X Last stage | 0.262***  (0.153,0.447) |  | 0.782  (0.360,1.700) |  | 0.942  (0.493,1.798) |  |
| Q5 X Delta | 0.622  (0.358,1.081) |  | 0.817  (0.399,1.673) |  | 1.218  (0.609,2.438) |  |
| Q5 X Omicron | 0.165***  (0.097,0.280) |  | 0.338***  (0.178,0.643) |  | 0.835  (0.453,1.536) |  |
| Q5 X Last stage | 0.212***  (0.124,0.361) |  | 0.646  (0.178,0.643) |  | 0.954  (0.515,1.765) |  |

Source: Authors’ own analysis of data from the 2020-23 Korean National Health Insurance Service (NHIS). Notes: Each column presents the results from a multivariate logistic model. The reference category consists of medical aid recipients, representing the lowest income group. Q1 through Q5 correspond to the first through fifth income quintiles, respectively. The analyses include 2,599,392 cases. * p<0.05, ** p<0.01, *** p<0.001.

Appendix Table 3. Results from logistic regression for cases younger than age 65

|  | Hospitalization | | Severe cases | | Fatalities | |
| --- | --- | --- | --- | --- | --- | --- |
|  | Model 1 | Model 2 | Model 1 | Model 2 | Model 1 | Model 2 |
|  | OR  (95% CI) | OR  (95% CI) | OR  (95% CI) | OR  (95% CI) | OR  (95% CI) | OR  (95% CI) |
| Income (ref.: Medical aid recipients) |  |  |  |  |  |  |
| Q1 | 0.491***  (0.469,0.514) | 1.698**  (1.149,2.508) | 0.885  (0.663,1.181) | 2.096  (0.869,5.057) | 0.498***  (0.453,0.548) | 0.657  (0.266,1.622) |
| Q2 | 0.468***  (0.447,0.490) | 1.575*  (1.069,2.319) | 0.821  (0.614,1.099) | 1.862  (0.768,4.518) | 0.436***  (0.395,0.482) | 0.603  (0.244,1.488) |
| Q3 | 0.443***  (0.423,0.463) | 1.404  (0.955,2.063) | 0.731*  (0.549,0.973) | 2.539*  (1.069,6.032) | 0.404***  (0.367,0.445) | 0.554  (0.227,1.351) |
| Q4 | 0.423***  (0.404,0.442) | 1.553*  (1.056,2.282) | 0.756  (0.569,1.006) | 2.201  (0.928,5.219) | 0.341***  (0.310,0.376) | 0.439  (0.179,1.078) |
| Q5 | 0.391***  (0.374,0.409) | 1.691**  (1.151,2.483) | 0.810  (0.613,1.070) | 2.590*  (1.104,6.076) | 0.294***  (0.267,0.324) | 0.202**  (0.076,0.537) |
| Female | 0.986  (0.971,1.001) | 0.986  (0.971,1.001) | 0.542***  (0.486,0.606) | 0.544***  (0.487,0.607) | 0.401***  (0.384,0.420) | 0.401***  (0.384,0.420) |
| Employed | 0.710***  (0.699,0.721) | 0.710***  (0.699,0.721) | 0.567***  (0.508,0.632) | 0.569***  (0.510,0.634) | 0.402***  (0.384,0.420) | 0.402***  (0.384,0.420) |
| Metropolitan area | 0.624***  (0.615,0.634) | 0.626***  (0.616,0.636) | 1.290***  (1.126,1.478) | 1.298***  (1.133,1.488) | 0.812***  (0.777,0.849) | 0.812***  (0.777,0.849) |
| Disability | 2.560***  (2.479,2.645) | 2.515***  (2.435,2.598) | 4.383***  (3.733,5.145) | 4.338***  (3.692,5.098) | 2.770***  (2.577,2.978) | 2.772***  (2.579,2.980) |
| Vaccination | 0.299***  (0.293,0.305) | 0.299***  (0.293,0.305) | 0.078***  (0.068,0.090) | 0.079***  (0.069,0.091) | 0.371***  (0.347,0.397) | 0.371***  (0.347,0.397) |
| Comorbidity | 1.515***  (1.492,1.539) | 1.515***  (1.492,1.539) | 3.717***  ([3.341,4.135] | 3.708***  (3.333,4.125) | 2.827***  (2.708,2.951) | 2.826***  (2.707,2.950) |
| COVID-19 Stage (ref.: Initial stage) |  |  |  |  |  |  |
| Delta | 0.258***  (0.247,0.270) | 0.338***  (0.227,0.503) | 1.771***  (1.543,2.032) | 3.977**  (1.576,10.035) | 1.714***  (1.349,2.177) | 2.080  (0.868,4.987) |
| Omicron | 0.003***  (0.003,0.003) | 0.013***  (0.009,0.018) | 0.093***  (0.078,0.111) | 0.407*  (0.168,0.986) | 1.517***  (1.224,1.880) | 1.855  (0.851,4.045) |
| Last stage | 0.004***  (0.003,0.004) | 0.012***  (0.008,0.017) | 0.101***  (0.082,0.124) | 0.312*  (0.113,0.864) | 1.646***  (1.325,2.045) | 1.743  (0.794,3.828) |
| Income X COVID-19 stage |  |  |  |  |  |  |
| Q1 X Delta |  | 0.738  (0.487,1.118) |  | 0.478  (0.177,1.293) |  | 0.692  (0.246,1.948) |
| Q1 X Omicron |  | 0.257***  (0.173,0.380) |  | 0.324*  (0.124,0.844) |  | 0.723  (0.292,1.791) |
| Q1 X Last stage |  | 0.332***  (0.223,0.494) |  | 0.461  (0.149,1.427) |  | 0.872  (0.348,2.184) |
| Q2 X Delta |  | 0.822  (0.544,1.241) |  | 0.608  (0.225,1.641) |  | 0.709  (0.252,1.990) |
| Q2 X Omicron |  | 0.261***  (0.177,0.386) |  | 0.305*  (0.116,0.801) |  | 0.690  [0.279,1.708] |
| Q2 X Last stage |  | 0.321***  (0.216,0.477) |  | 0.264*  (0.080,0.871) |  | 0.819  (0.328,2.050) |
| Q3 X Delta |  | 0.870  (0.578,1.310) |  | 0.349*  (0.132,0.925) |  | 0.629  (0.227,1.747) |
| Q3 X Omicron |  | 0.278***  (0.189,0.410) |  | 0.172***  (0.067,0.443) |  | 0.712  (0.291,1.740) |
| Q3 X Last stage |  | 0.342***  (0.231,0.507) |  | 0.200**  (0.064,0.628) |  | 0.784  (0.317,1.936) |
| Q4 X Delta |  | 0.760  (0.505,1.145) |  | 0.407  (0.154,1.075) |  | 0.750  (0.270,2.086) |
| Q4 X Omicron |  | 0.237***  (0.161,0.349) |  | 0.210**  (0.082,0.535) |  | 0.749  (0.304,1.843) |
| Q4 X Last stage |  | 0.297***  (0.200,0.440) |  | 0.396  (0.133,1.179) |  | 0.859  (0.346,2.134) |
| Q5 X Delta |  | 0.676  (0.449,1.018) |  | 0.430  (0.165,1.122) |  | 1.647  (0.554,4.902) |
| Q5 X Omicron |  | 0.199***  (0.135,0.293] |  | 0.155***  (0.061,0.391) |  | 1.376  (0.517,3.658) |
| Q5 X Last stage |  | 0.240***  (0.162,0.356) |  | 0.266*  (0.090,0.788) |  | 1.705  (0.636,4.573) |

Source: Authors’ own analysis of data from the 2020-23 Korean National Health Insurance Service (NHIS). Notes: Each column presents the results from a multivariate logistic model. The reference category consists of medical aid recipients, representing the lowest income group. Q1 through Q5 correspond to the first through fifth income quintiles, respectively. The analyses include 4,830,509 cases. * p<0.05, ** p<0.01, *** p<0.001.

Appendix Table 4. Results from logistic regression for cases at age 65 or older

|  | Hospitalization | | Severe cases | | Fatalities | |
| --- | --- | --- | --- | --- | --- | --- |
|  | Model 1 | Model 2 | Model 1 | Model 2 | Model 1 | Model 2 |
|  | OR  (95% CI) | OR  (95% CI) | OR  (95% CI) | OR  (95% CI) | OR  (95% CI) | OR  (95% CI) |
| Income (ref.: Medical aid recipients) |  |  |  |  |  |  |
| Q1 | 0.677***  (0.649,0.707) | 0.987  (0.224,4.336) | 0.921  (0.773,1.096) | 1.626  (0.929,2.849) | 0.846***  (0.806,0.889) | 1.623  (0.911,2.892) |
| Q2 | 0.563***  (0.538,0.589) | 0.741  (0.167,3.292) | 0.765**  (0.638,0.918) | 0.922  (0.504,1.687) | 0.577***  (0.547,0.608) | 0.508  (0.253,1.017) |
| Q3 | 0.529***  (0.507,0.552) | 1.210  (0.255,5.753) | 0.675***  (0.566,0.806) | 0.596  (0.318,1.119) | 0.515***  (0.489,0.541) | 0.586  (0.306,1.121) |
| Q4 | 0.507***  (0.487,0.528) | 0.566  (0.131,2.434) | 0.686***  (0.581,0.811) | 0.862  (0.488,1.520) | 0.425***  (0.405,0.446) | 0.556  (0.303,1.020) |
| Q5 | 0.514***  (0.494,0.534) | 1.117  (0.255,4.900) | 0.696***  (0.594,0.815) | 0.862  (0.501,1.482) | 0.451***  (0.431,0.471) | 0.640  (0.365,1.123) |
| Female | 0.868*** (0.852,0.884) | 0.867*** (0.852,0.884) | 0.428*** (0.396,0.462) | 0.428*** (0.396,0.462) | 0.500*** (0.489,0.511) | 0.500** (0.365,1.123) |
| Employed | 0.486***  (0.471,0.501) | 0.484***  (0.470,0.500) | 0.436***  (0.384,0.494) | 0.436***  (0.384,0.494) | 0.226***  (0.215,0.237) | 0.226***  (0.215,0.237) |
| Metropolitan area | 0.589***  (0.578,0.600) | 0.589***  (0.578,0.600) | 1.244***  (1.144,1.352) | 1.243***  (1.144,1.351) | 0.680***  (0.665,0.696) | 0.680***  (0.665,0.696) |
| Disability | 2.004***  (1.960,2.049) | 2.002***  (0.578,0.600) | 2.113***  (1.936,2.307) | 2.113***  (1.936,2.307) | 1.980***  (1.928,2.033) | 1.980***  (1.928,2.033) |
| Vaccination | 0.262***  (0.253,0.272) | 0.262***  (0.253,0.272) | 0.110***  (0.099,0.121) | 0.110***  (0.099,0.121) | 0.252***  (0.242,0.263) | 0.252***  (0.242,0.263) |
| Comorbidity | 1.437***  (1.403,1.471) | 1.437***  (1.403,1.471) | 1.488***  (1.350,1.641) | 1.489***  (1.351,1.643) | 1.520***  (1.474,1.567) | 1.520***  (1.474,1.567) |
| COVID-19 Stage (ref.: Initial stage) |  |  |  |  |  |  |
| Delta | 0.103***  (0.082,0.129) | 0.0862***  (0.021,0.351) | 3.323***  (2.865,3.854) | 3.783***  (2.100,6.815) | 3.357***  (2.871,3.925) | 4.433***  (2.523,7.791) |
| Omicron | 0.00189***  (0.002,0.002) | 0.00313***  (0.001,0.013) | 0.216***  (0.185,0.252) | 0.330***  (0.193,0.563) | 2.233***  (1.936,2.576) | 3.047***  (1.828,5.078) |
| Last stage | 0.00230***  (0.002,0.003) | 0.00332***  (0.001,0.013) | 0.231***  (0.196,0.272) | 0.205***  (0.110,0.382) | 2.448***  (2.121,2.826) | 3.221***  (1.927,5.386) |
| Income X COVID-19 stage |  |  |  |  |  |  |
| Q1 X Delta |  | 1.326  (0.298,5.900) |  | 0.512*  (0.263,0.999) |  | 0.477*  (0.249,0.914) |
| Q1 X Omicron |  | 0.645  (0.147,2.836) |  | 0.446**  (0.245,0.813) |  | 0.517*  (0.249,0.914) |
| Q1 X Last stage |  | 0.738  (0.168,3.251) |  | 0.933  (0.466,1.871) |  | 0.527*  (0.294,0.946) |
| Q2 X Delta |  | 1.466  (0.325,6.604) |  | 0.921  (0.452,1.877) |  | 1.101  (0.513,2.364) |
| Q2 X Omicron |  | 0.711  (0.160,3.163) |  | 0.680  (0.355,1.301) |  | 1.121  (0.558,2.252) |
| Q2 X Last stage |  | 0.823  (0.185,3.665) |  | 1.232  (0.582,2.608) |  | 1.184  (0.587,2.388) |
| Q3 X Delta |  | 0.824  (0.171,3.966) |  | 1.385  (0.668,2.872) |  | 0.902  (0.439,1.850) |
| Q3 X Omicron |  | 0.403  (0.085,1.920) |  | 0.920  (0.471,1.799) |  | 0.851  (0.443,1.634) |
| Q3 X Last stage |  | 0.489  (0.103,2.330) |  | 1.711  (0.797,3.677) |  | 0.945  (0.490,1.820) |
| Q4 X Delta |  | 1.821  (0.418,7.938) |  | 0.952  (0.487,1.858) |  | 0.769  (0.391,1.514) |
| Q4 X Omicron |  | 0.822  (0.191,3.541) |  | 0.650  (0.354,1.193) |  | 0.761  (0.413,1.401) |
| Q4 X Last stage |  | 1.002  (0.232,4.319) |  | 1.056  (0.522,2.135) |  | 0.770  (0.416,1.422) |
| Q5 X Delta |  | 0.824  (0.186,3.659) |  | 0.932  (0.492,1.766) |  | 0.792  (0.423,1.481) |
| Q5 X Omicron |  | 0.436  (0.099,1.914) |  | 0.661  (0.371,1.179) |  | 0.694  (0.394,1.220) |
| Q5 X Last stage |  | 0.491  (0.112,2.160) |  | 1.130  (0.578,2.208) |  | 0.718  (0.407,1.267) |

Source: Authors’ own analysis of data from the 2020-23 Korean National Health Insurance Service (NHIS). Notes: Each column presents the results from a multivariate logistic model. The reference category consists of medical aid recipients, representing the lowest income group. Q1 through Q5 correspond to the first through fifth income quintiles, respectively. The analyses include 1,032,749 cases. * p<0.05, ** p<0.01, *** p<0.001.

Appendix Table 5. Results from logistic regression for the employed

|  | Hospitalization | | Severe cases | | Fatalities | |
| --- | --- | --- | --- | --- | --- | --- |
|  | Model 1 | Model 2 | Model 1 | Model 2 | Model 1 | Model 2 |
|  | OR  (95% CI) | OR  (95% CI) | OR  (95% CI) | OR  (95% CI) | OR  (95% CI) | OR  (95% CI) |
| Income (ref.: Q1) |  |  |  |  |  |  |
| Q2 | 0.921***  (0.894,0.948) | 0.903  (0.753,1.083) | 0.917  (0.754,1.117) | 0.757  (0.484,1.186) | 0.802***  (0.741,0.869) | 0.665  (0.326,1.354) |
| Q3 | 0.871***  (0.845,0.898) | 0.662***  (0.557,0.786) | 0.745**  (0.604,0.920) | 0.845  (0.543,1.317) | 0.751***  (0.690,0.817) | 0.780  (0.396,1.535) |
| Q4 | 0.827***  (0.801,0.853) | 0.838  (0.701,1.001) | 0.778*  (0.627,0.964) | 1.023  (0.675,1.549) | 0.644***  (0.589,0.704) | 0.648  (0.325,1.293) |
| Q5 | 0.776***  (0.751,0.801) | 0.908  (0.760,1.085) | 1.031  (0.840,1.265) | 1.392  (0.955,2.028) | 0.612***  (0.560,0.669) | 0.463*  (0.223,0.958) |
| Age (ref.: 65>) |  |  |  |  |  |  |
| 65-84 | 1.839***  (1.779,1.901) | 1.828***  (1.769,1.889) | 5.590***  (4.707,6.638) | 5.581***  (4.696,6.632) | 4.419***  (4.115,4.746) | 4.419***  (4.115,4.746) |
| 85<= | 1.839***  (1.779,1.901) | 8.568***  (6.782,10.825) | 37.652***  (17.567,80.703) | 37.608***  (17.585,80.432) | 35.195***  (27.591,44.895) | 35.206***  (27.600,44.907) |
| Female | 1.007  (0.986,1.027) | 1.006  (0.985,1.026) | 0.446***  (0.381,0.522) | 0.447***  (0.382,0.523) | 0.346***  (0.324,0.369) | 0.346***  (0.324,0.369) |
| Metropolitan area | 0.634***  (0.621,0.647) | 0.635***  (0.622,0.648) | 1.507***  (1.256,1.807) | 1.510***  (1.259,1.811) | 0.862***  (0.814,0.913) | 0.862***  (0.814,0.913) |
| Disability | 2.063***  (1.970,2.161) | 2.056***  (1.964,2.154) | 3.064***  (2.488,3.775) | 3.063***  (2.489,3.770) | 2.222***  (2.034,2.428) | 2.223***  (2.034,2.429) |
| Vaccination | 0.285***  (0.278,0.293) | 0.286***  (0.278,0.293) | 0.088***  (0.073,0.105) | 0.088***  (0.073,0.105) | 0.420***  (0.377,0.468) | 0.419***  (0.376,0.467) |
| Comorbidity | 1.420***  (1.392,1.450) | 1.420***  (1.391,1.449) | 2.649***  (2.289,3.066) | 2.640***  (2.280,3.057) | 2.214***  (2.083,2.353) | 2.214***  (2.083,2.353) |
| COVID-19 Stage (ref.: Initial stage) |  |  |  |  |  |  |
| Delta | 0.263***  (0.248,0.279) | 0.227***  (0.197,0.262) | 2.064***  (1.740,2.449) | 2.271***  (1.609,3.207) | 1.650***  (1.254,2.171) | 1.663*  (1.015,2.725) |
| Omicron | 0.003***  (0.003,0.003) | 0.003***  (0.002,0.003) | 0.069***  (0.054,0.088) | 0.086***  (0.058,0.128) | 0.975  (0.755,1.259) | 0.902  (0.581,1.400) |
| Last stage | 0.003***  (0.003,0.003) | 0.003***  (0.003,0.004) | 0.075***  (0.057,0.100) | 0.091***  (0.055,0.151) | 1.036  (0.799,1.342) | 0.967  (0.618,1.512) |
| Income X COVID-19 stage |  |  |  |  |  |  |
| Q2 X Delta |  | 1.172  (0.967,1.420) |  | 1.292  (0.757,2.205) |  | 1.103  (0.487,2.497) |
| Q2 X Omicron |  | 0.999  (0.830,1.203) |  | 1.241  (0.700,2.200) |  | 1.198  (0.585,2.456) |
| Q2 X Last stage |  | 0.941  (0.776,1.141) |  | 1.208  (0.562,2.598) |  | 1.262  (0.609,2.616) |
| Q3 X Delta |  | 1.487***  (1.239,1.785) |  | 0.938  (0.551,1.597) |  | 0.815  (0.369,1.800) |
| Q3 X Omicron |  | 1.332**  (1.117,1.588) |  | 0.751  (0.413,1.366) |  | 0.988  (0.499,1.956) |
| Q3 X Last stage |  | 1.173  (0.976,1.409) |  | 0.714  (0.308,1.654) |  | 0.911  (0.454,1.829) |
| Q4 X Delta |  | 1.131  (0.937,1.365) |  | 0.624  (0.372,1.047) |  | 0.876  (0.389,1.972) |
| Q4 X Omicron |  | 0.969  (0.808,1.163) |  | 0.789  (0.456,1.367) |  | 1.001  (0.499,2.008) |
| Q4 X Last stage |  | 0.896  (0.741,1.082) |  | 0.853  (0.405,1.797) |  | 0.991  (0.487,2.013) |
| Q5 X Delta |  | 1.031  (0.854,1.244) |  | 0.844  (0.534,1.335) |  | 1.237  (0.532,2.877) |
| Q5 X Omicron |  | 0.836  (0.697,1.003) |  | 0.462**  (0.272,0.784) |  | 1.336  (0.643,2.776) |
| Q5 X Last stage |  | 0.714***  (0.591,0.863) |  | 0.565  (0.275,1.161) |  | 1.317  (0.627,2.768) |

Source: Authors’ own analysis of data from the 2020-23 Korean National Health Insurance Service (NHIS). Notes: Each column presents the results from a multivariate logistic model. The reference category consists of medical aid recipients, representing the lowest income group. Q1 through Q5 correspond to the first through fifth income quintiles, respectively. The analyses include 3,412,564 cases. * p<0.05, ** p<0.01, *** p<0.001.

Appendix Table 6. Results from logistic regression for the unemployed

|  | Hospitalization | | Severe cases | | Fatalities | |
| --- | --- | --- | --- | --- | --- | --- |
|  | Model 1 | Model 2 | Model 1 | Model 2 | Model 1 | Model 2 |
|  | OR  (95% CI) | OR  (95% CI) | OR  (95% CI) | OR  (95% CI) | OR  (95% CI) | OR  (95% CI) |
| Income (ref.: Medical aid recipients) |  |  |  |  |  |  |
| Q1 | 0.558***  (0.540,0.577) | 1.004  (0.678,1.488) | 0.837*  (0.738,0.858) | 1.334  (0.718,0.976) | 0.656***  (0.627,0.686) | 1.068  (0.626,1.820) |
| Q2 | 0.521***  (0.504,0.539) | 0.897  (0.609,1.321) | 0.741***  (0.678,0.798) | 0.994  (0.631,0.870) | 0.518***  (0.493,0.545) | 0.494*  (0.267,0.916) |
| Q3 | 0.496***  (0.480,0.512) | 1.245  (0.845,1.834) | 0.676***  (0.628,0.733) | 1.073  (0.580,0.788) | 0.472***  (0.451,0.495) | 0.494*  (0.275,0.887) |
| Q4 | 0.486***  (0.472,0.501) | 1.082  (0.741,1.580) | 0.695***  (0.590,0.683) | 1.001  (0.601,0.803) | 0.407***  (0.389,0.425) | 0.463**  (0.267,0.801) |
| Q5 | 0.467***  (0.453,0.481) | 1.311  (0.900,1.910) | 0.662***  (0.581,0.663) | 0.950  (0.577,0.760) | 0.391***  (0.376,0.408) | 0.446**  (0.267,0.747) |
| Age (ref.: 65>) |  |  |  |  |  |  |
| 65-84 | 2.088***  (2.052,2.125) | 2.091***  (2.055,2.128) | 5.415***  (22.787,25.911) | 5.415***  (4.888,5.997) | 6.296***  (6.079,6.526) | 6.296***  (6.077,6.523) |
| 85<= | 6.206***  (6.018,6.400) | 6.203***  (6.014,6.397) | 15.590***  (62.795,72.386) | 15.463***  (13.479,18.032) | 45.121***  (43.292,47.077) | 45.121***  (43.269,47.051) |
| Female | 0.871***  (0.858,0.884) | 0.870***  (0.858,0.883) | 0.447***  (0.516,0.558) | 0.447***  (0.417,0.480) | 0.450***  (0.440,0.460) | 0.450***  (0.440,0.460) |
| Metropolitan area | 0.612***  (0.603,0.621) | 0.612***  (0.603,0.621) | 1.271***  (1.500,1.641) | 1.271***  (1.175,1.376) | 0.745***  (0.729,0.762) | 0.745***  (0.729,0.762) |
| Disability | 2.092***  (2.050,2.135) | 2.085***  (2.043,2.128) | 2.240***  (2.387,2.626) | 2.237***  (2.055,2.442) | 1.854***  (1.804,1.904) | 1.854***  (1.804,1.905) |
| Vaccination | 0.298***  (0.292,0.305) | 0.299***  (0.292,0.305) | 0.114***  (0.161,0.178) | 0.114***  (0.104,0.124) | 0.292***  (0.280,0.303) | 0.291***  (0.280,0.303) |
| Comorbidity | 1.543***  (1.517,1.570) | 1.543***  (1.517,1.570) | 2.258***  (2.039,2.499) | 2.255***  (2.037,2.496) | 1.785***  (1.730,1.842) | 1.785***  (1.729,1.842) |
| COVID-19 Stage (ref.: Initial stage) |  |  |  |  |  |  |
| Delta | 0.229***  (0.214,0.245) | 0.271***  (0.187,0.393) | 2.612***  (1.787,2.131) | 3.638***  (2.291,2.978) | 2.789***  (2.401,3.239) | 3.328***  (2.009,5.515) |
| Omicron | 0.004***  (0.004,0.004) | 0.009***  (0.007,0.014) | 0.177***  (0.147,0.174) | 0.302***  (0.154,0.204) | 1.956***  (1.705,2.243) | 2.325***  (1.474,3.669) |
| Last stage | 0.005***  (0.004,0.005) | 0.009***  (0.007,0.013) | 0.188***  (0.149,0.179) | 0.195***  (0.162,0.218) | 2.096***  (1.825,2.406) | 2.326***  (1.470,3.681) |
| Income X COVID-19 stage |  |  |  |  |  |  |
| Q1 X Delta |  | 0.964  (0.638,1.456) |  | 0.447**  (0.243,0.822) |  | 0.519*  (0.284,0.950) |
| Q1 X Omicron |  | 0.519**  (0.350,0.771) |  | 0.554*  (0.319,0.962) |  | 0.605  (0.354,1.034) |
| Q1 X Last stage |  | 0.618*  (0.415,0.920) |  | 1.101  (0.585,2.070) |  | 0.638  (0.372,1.094) |
| Q2 X Delta |  | 1.027  (0.685,1.542) |  | 0.765  (0.412,1.423) |  | 0.920  (0.464,1.824) |
| Q2 X Omicron |  | 0.545**  (0.369,0.805) |  | 0.644  (0.362,1.147) |  | 1.030  (0.555,1.914) |
| Q2 X Last stage |  | 0.645*  (0.435,0.955) |  | 0.988  (0.504,1.935) |  | 1.113  (0.597,2.077) |
| Q3 X Delta |  | 0.763  (0.508,1.144) |  | 0.654  (0.360,1.188) |  | 0.879  (0.459,1.683) |
| Q3 X Omicron |  | 0.358***  (0.243,0.529) |  | 0.511*  (0.294,0.888) |  | 0.925  (0.514,1.663) |
| Q3 X Last stage |  | 0.457***  (0.309,0.676) |  | 0.864  (0.455,1.640) |  | 1.045  (0.579,1.887) |
| Q4 X Delta |  | 0.922  (0.620,1.370) |  | 0.796  (0.450,1.407) |  | 0.823  (0.446,1.519) |
| Q4 X Omicron |  | 0.401***  (0.274,0.587) |  | 0.547*  (0.323,0.928) |  | 0.865  (0.499,1.502) |
| Q4 X Last stage |  | 0.510***  (0.347,0.747) |  | 0.935  (0.508,1.721) |  | 0.914  (0.525,1.593) |
| Q5 X Delta |  | 0.718  (0.485,1.064) |  | 0.771  (0.445,1.336) |  | 1.012  (0.570,1.796) |
| Q5 X Omicron |  | 0.323*** (0.221,0.471) |  | 0.567*  (0.342,0.941) |  | 0.853  (0.509,1.431) |
| Q5 X Last stage |  | 0.390***  (0.267,0.571) |  | 0.946  (0.528,1.696) |  | 0.925  (0.550,1.556) |

Source: Authors’ own analysis of data from the 2020-23 Korean National Health Insurance Service (NHIS). Notes: Each column presents the results from a multivariate logistic model. The reference category consists of medical aid recipients, representing the lowest income group. Q1 through Q5 correspond to the first through fifth income quintiles, respectively. The analyses include 2,450,694 cases. * p<0.05, ** p<0.01, *** p<0.001.
